# Biomarker validation of cardiac magnetic resonance analysis of regional myocardial fibrosis in ischaemic heart disease

**DOI:** 10.1186/1532-429X-18-S1-P77

**Published:** 2016-01-27

**Authors:** Benedict T Costello, Leah M Iles, Dion Stub, Andris Ellims, Karen Smith, Stephen Bernard, Ziad Nehme, Janet Bray, Peter Cameron, Ian Meredith, David Kaye, Andrew Taylor

**Affiliations:** 1 grid.1051.50000000097605620Baker IDI Heart and Diabetes Institute, Melbourne, VIC Australia; 2 Alfred Hospital, Melbourne, VIC Australia; 3 grid.1002.30000000419367857Monash University, Melbourne, VIC Australia; 4 Ambulance Victoria, Melbourne, VIC Australia; 5 Monash Heart, Melbourne, VIC Australia

## Background

Late gadolinium enhancement (LGE) with CMR is commonly assumed to represent myocardial fibrosis; however, comparative human histological data are limited, and there is no consensus on the most accurate method for LGE quantitation. We evaluated the relationship between CMR assessment of regional fibrosis and infarct size assessment using serial biomarkers after ST segment elevation myocardial infarction (STEMI).

## Methods

Ninety-five patients treated for STEMI (59 ± 10 years, 85% male) underwent CMR six months after infarction. Fibrosis was quantified by CMR-LGE using visual and automated thresholds, and compared with the rise in serum biomarkers.

## Results

Quantification methods had a strong influence on the infarct size assessment with CMR-LGE. Significant correlations were observed between LGE and biomarkers across a range of signal intensity thresholds (range: 2-10 standard deviations [SD] above reference myocardium), however there was a wide range with respect to estimation of total LGE size (from 6.8 ± 7.7 to 32.1 ± 11.3 grams) and a smaller variation in the correlation with peak troponin level (R-values ranging from 0.715 to 0.834). The strongest correlation was observed at thresholds of 5 and 6 SD (R = 0.830, P < 0.001 and R = 0.834, P < 0.001).

## Conclusions

There is a wide variation for the correlation between CMR-LGE quantification of infarct size and biomarker release following STEMI at a range of automated thresholds, with the strongest correlations at 5SD and 6SD thresholds.

**Figure 1:**
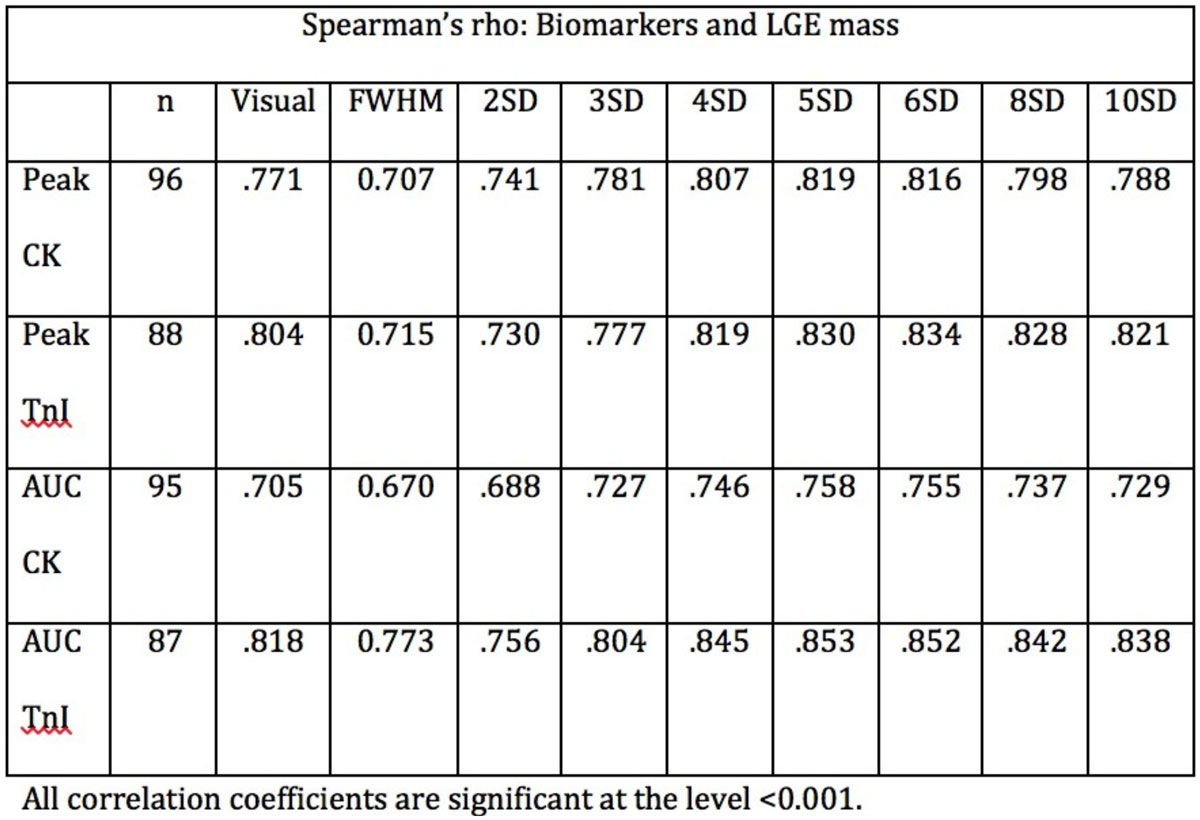
**Correlation between infarct size assessment using biomarkers and CMR-LGE**.

